# Parent-reported measures of child health and wellbeing in same-sex parent families: a cross-sectional survey

**DOI:** 10.1186/1471-2458-14-635

**Published:** 2014-06-21

**Authors:** Simon R Crouch, Elizabeth Waters, Ruth McNair, Jennifer Power, Elise Davis

**Affiliations:** 1 The Jack Brockhoff Child Health and Wellbeing Program, The Melbourne School of Population and Global Health, The University of Melbourne, Carlton, Victoria, Australia; 2 The Department of General Practice, The University of Melbourne, Carlton, Victoria, Australia; 3 The Bouverie Centre, La Trobe University, Brunswick, Victoria, Australia

**Keywords:** Child health, Stigma, Sexuality, Parenting

## Abstract

**Background:**

It has been suggested that children with same-sex attracted parents score well in psychosocial aspects of their health, however questions remain about the impact of stigma on these children. Research to date has focused on lesbian parents and has been limited by small sample sizes. This study aims to describe the physical, mental and social wellbeing of Australian children with same-sex attracted parents, and the impact that stigma has on them.

**Methods:**

A cross-sectional survey, the Australian Study of Child Health in Same-Sex Families, was distributed in 2012 to a convenience sample of 390 parents from Australia who self-identified as same-sex attracted and had children aged 0-17 years. Parent-reported, multidimensional measures of child health and wellbeing and the relationship to perceived stigma were measured.

**Results:**

315 parents completed the survey (completion rate = 81%) representing 500 children. 80% of children had a female index parent while 18% had a male index parent. Children in same-sex parent families had higher scores on measures of general behavior, general health and family cohesion compared to population normative data (β = 2.93, 95% CI = 0.35 to 5.52, P = .03; β = 5.60, 95% CI = 2.69 to 8.52, P = <.001; and β = 6.01, 95% CI = 2.84 to 9.17, P = <.001 respectively). There were no significant differences between the two groups for all other scale scores. Physical activity, mental health, and family cohesion were all negatively associated with increased stigma (β = -3.03, 95% CI = -5.86 to -0.21, P = .04; β = -10.45, 95% CI = -18.48 to -2.42, P = .01; and β = -9.82, 95% CI = -17.86 to -1.78, P = .02 respectively) and the presence of emotional symptoms was positively associated with increased stigma (β =0.94, 95% CI = 0.08 to 1.81, P = .03).

**Conclusions:**

Australian children with same-sex attracted parents score higher than population samples on a number of parent-reported measures of child health. Perceived stigma is negatively associated with mental health. Through improved awareness of stigma these findings play an important role in health policy, improving child health outcomes.

## Background

It is estimated that in 2011 there were 6,120 children under the age of 25 years living with two same-sex parents in Australia [1], with the number of same-sex couple households increasing from around 19,000 to more than 33,000 over the preceding ten years [1,2]. These figures are a conservative estimate as they do not capture children living with same-sex attracted single parents, or parents who are reluctant to self-identify as same-sex attracted due to fear of stigma and discrimination. Ongoing reforms in Australia around same-sex adoption, surrogacy and fertility treatments will only see this number rise, with a recent national survey on the health and wellbeing of gay, lesbian, bisexual and transgender Australians identifying that 22.1% of respondents have children or step children [3].

Two decades of research from Northern Europe and the United States suggests that the health and wellbeing of children with same-sex attracted parents is no different when compared to children from other family backgrounds, particularly in relation to social and emotional development and educational outcomes [4-6]. Stacey and Biblarz (2001) argue however that a more detailed consideration of the literature identifies a number of areas that do not necessarily follow this commonly acknowledged ‘no difference’ consensus [7]. This includes a focus on sexual orientation, although it is now generally accepted that child sexual orientation is not a measure of quality parenting [7]. In fact, a number of authors agree that simply asking a question that compares the sexual orientation of children with same-sex parents to children with heterosexual parents reinforces a heterosexist viewpoint that stigmatises same-sex families [8].

What the research is beginning to show however is the importance of such stigmatisation, as it is a key factor that impacts on the health and wellbeing of children with same-sex attracted parents [4,9-15]. Numerous studies have found that when there is perceived stigma, experienced rejection or homophobic bullying, children with same-sex attracted parents are more likely to display problems in their psychosocial development [16-22]. These experiences and their impacts differ globally with children from the US experiencing more homophobia, and associated higher levels of problem behaviour, when compared to children from the Netherlands [17]. The only Australian study to date to consider these issues identified high levels of bullying toward children with same-sex attracted parents but did not consider health outcomes [21].

Previous research has taken a narrow perspective when considering broader aspects of health, limiting data to a few common childhood ailments [23]. Prevention, early intervention, continuity of care and integration of healthcare services are particularly important for child health during early years [24] and it has been suggested that lesbian parents perceive barriers when dealing with the healthcare system in Australia [25]. As such it is important to determine whether potential barriers, such as perceived stigma, have an impact on the physical wellbeing of children with same-sex attracted parents.

When considering child health in same-sex parent families, research on the role of parent gender is conflicted. While some studies suggest that mothers are more emotionally invested in raising children than fathers are in general [26-28], further work suggests that absent fathers may be detrimental to self-rated cognitive and physical competence [29]. Regardless of gender however it is becoming clear that same-sex attracted parents construct their parenting roles more equitably than heterosexual parents and this may be of benefit to family functioning [30]. With this in mind it should be noted that there is a lack of research looking at male same-sex parented families [31], and too often authors extrapolate results from research on lesbian parenting to the whole range of same-sex families [32]. When studies have looked at gay male parents it is usually in the context of a kinship arrangement where the father acts as a sperm donor [33].

Studies to date have often relied on small samples. Such sample sizes limit statistical analysis and the wider application of findings to the broader community. Convenience samples are also commonly used and are often fraught with problems. As participants are self-selecting such studies are open to accusations of bias that might skew results in favour of same-sex parent families and capture only specific subsets of the gay and lesbian community [34]. It is not clear whether such bias is at play in previous samples however, but by employing recruitment strategies that actively seek to reach a broad range of families a more representative sample can be achieved [35]. In some contexts researchers have aimed to overcome limitations of convenience sampling by extrapolating same-sex attracted parents from population surveys [36]. In such cases many assumptions are made as same-sex attraction is presumed based on other demographic characteristics (such as children indicating two parents of the same gender). This limits data to same-sex couple families and does not allow for a broader representation of the community, such as single same-sex attracted parents and co-parenting arrangements to name but two [37]. In Australia current political and cultural discourse does not allow the capturing of sexual orientation in national population surveys. Although household surveys may be of benefit, cost constraints and likely small sample sizes make them impractical and as such the only methodology available to capture child health in the context of same-sex parent families is through convenience sampling. By utilising strategic and broad ranging recruitment techniques however, the best available study population can be achieved under difficult research constraints.

The overall aim of this study was to understand the multidimensional experiences of physical, mental and social wellbeing of children in same-sex parent family contexts, in addition to providing a contemporary policy relevant profile of the diversity and complexity of families and their social and physical environments [38]. The objectives were: to describe the characteristics of a convenience sample of families with at least one same-sex attracted parent; to measure the physical, mental and social wellbeing of children living in this context; and to determine the relationship between perceived stigma and child health and wellbeing.

## Methods

The full methodology for the Australian Study of Child Health in Same-Sex Families is described in the study protocol [38], while the methods relating to the results presented here are summarised below.

### Study participants and data collection

The study, named the Australian Study of Child Health in Same-Sex Families (ACHESS), was conducted throughout Australia using a confidential cross-sectional survey to collect data between May and December 2012. Strategies were employed to contact same-sex attracted parents who both identified with the gay and lesbian community, and those who were less engaged [35]. The survey was available to complete online and in paper form. Data was collected from index parents who self-identified as being same-sex attracted, were residing in Australia, and were over the age of 18 years. Parents reported information for all children under the age of 18 years. The convenience sample was recruited using online and traditional recruitment techniques, accessing same-sex attracted parents through news media, community events and community groups. Three hundred and ninety eligible parents contacted the researchers in the first instance with two reminders for non-completion. Population normative data was available for two of the survey instruments, the Child Health Questionnaire (from the Health of Young Victorians Survey, HOYVS) [39] and the Strengths and Difficulties Questionnaire (from the Victorian Child Health and Wellbeing Survey, VCHWS) [40].

### Survey instrument

Survey preparation comprised a scoping review of the literature [38], consultations with same-sex attracted parents and adult children with same-sex attracted parents. The survey was constructed around embedded, established, psychometrically validated and reliable measures of child health which included the Child Health Questionnaire (CHQ), the Infant Toddler Quality of Life survey (ITQOL), and the Strengths and Difficulties Questionnaire [38]. Standard demographic characteristics from population surveys and previous work with same-sex parent families were included [41,42]. The survey aimed to identify a contemporary picture of same-sex parent family socioeconomic contexts and family structures. Child health and wellbeing and perceived stigma were the two main outcome measures.

### Family structure and socioeconomic context

Parents were asked to report on their sexual orientation, current socioeconomic context, methods of family formation, and family structure as listed in Table 1.

**Table 1:** Child demographic characteristics

		**Number of children (%)**	
**Child demographic characteristics**	**All children (n = 500)**^**a**^	**With male parent/s (n = 91)**	**With female parent/s (n = 400)**
Gender			
Male	264 (53)	49 (54)	214 (54)
Female	230 (46)	40 (44)	182 (46)
Mean age, years	5.12	3.86	5.43
Median age, years	4	2	4
Age range, years	0-17	0-16	0-17
Geographical location			
Inner metropolitan	261 (52)	64 (70)	195 (49)
Outer metropolitan	123 (25)	10 (11)	106 (27)
Regional center	69 (14)	3 (3)	66 (17)
Rural	35 (7)	8 (9)	27 (7)
Remote	1 (<1)	0 (0)	1 (<1)
Other	8 (2)	3 (3)	5 (1)
State			
Victoria	244 (48)	41 (45)	196 (49)
New South Wales	89 (18)	26 (29)	63 (16)
Queensland	74 (15)	3 (3)	71 (18)
Western Australia	30 (6)	6 (7)	24 (6)
South Australia	26 (5)	6 (7)	18 (5)
Australian Capital Territory	23 (5)	6 (7)	17 (4)
Tasmania	14 (3)	3 (3)	11 (3)
Country of birth			
Australia	427 (85)	31 (34)	387 (97)
India	36 (7)	36 (40)	0 (0)
USA	21 (4)	20 (22)	1 (<1)
UK	6 (1)	0 (0)	6 (2)
New Zealand	2 (<1)	1 (1)	1 (<1)
Thailand	1 (<1)	1 (1)	0 (0)
Other	7 (1)	2 (2)	5 (1)
Language			
English only	444 (89)	65 (71)	374 (93)
Language other than English	56 (11)	26 (29)	26 (7)
Index parent’s highest level of education			
4 years high school	8 (2)	0 (0)	8 (2)
Year 12	28 (6)	7 (8)	21 (5)
Diploma or certificate	77 (15)	11 (12)	61 (15)
Undergraduate degree	133 (27)	33 (36)	98 (25)
Postgraduate degree	232 (46)	36 (40)	194 (49)
Other	19 (4)	4 (4)	15 (4)
Household income (AUD)			
$10,000-$19,000	13 (3)	0 (0)	13 (3)
$20,000-$29,999	25 (5)	4 (4)	16 (4)
$30,000-$59,999	56 (11)	4 (4)	50 (13)
$60,000-$99,999	112 (22)	11 (12)	101 (25)
$100,000-$149,999	129 (26)	23 (25)	106 (27)
$150,000-$249,999	95 (19)	22 (24)	71 (18)
$250,000 or more	70 (14)	27 (30)	43 (10)
Index parent’s sexual orientation			
Lesbian	344 (69)	-	337 (84)
Gay	92 (18)	85 (93)	7 (2)
Bisexual	41 (8)	1 (1)	40 (10)
Queer	11 (2)	2 (2)	9 (2)
Heterosexual	3 (1)	0 (0)	3 (1)
Other	9 (2)	3 (3)	4 (1)
Transgender parent	6 (1)	2 (2)	1 (<1)
Child relationship to index parent^b^			
Biological child	310 (62)	46 (51)	256 (64)
Non-biological child	123 (24)	18 (20)	104 (26)
Partner’s biological child	98 (20)	17 (19)	80 (20)
Fostered	13 (3)	8 (9)	5 (1)
Adopted	2 (<1)	2 (2)	0 (0)
Parent relationship status at time of conception, fostering or adoption			
Current relationship	347 (69)	66 (73)	279 (70)
Index parent’s previous heterosexual relationship	50 (10)	10 (11)	40 (10)
Index parent’s previous same-sex relationship	37 (7)	2 (2)	35 (9)
While index parent single	33 (7)	8 (9)	23 (6)
Partner’s previous heterosexual relationship	19 (4)	0 (0)	19 (5)
While partner single	2 (<1)	2 (2)	0 (0)
Other	5 (1)	0 (0)	2 (1)
Where child lives			
With index parent full time	411 (82)	70 (77)	335 (84)
With index parent part time	50 (10)	16 (18)	24 (6)
With another parent full time	12 (2)	3 (3)	9 (2)
Lives independently	1 (<1)	0 (0)	1 (<1)
Other	24 (5)	2 (2)	19 (5)
Index parent currently in a relationship	464 (93)	83 (91)	374 (94)
Method of conception^b^			
Heterosexual intercourse	102 (20)	18 (20)	79 (20)
Home insemination – known or own gametes	137 (27)	7 (8)	127 (32)
ART^c^ – unknown donor	148 (30)	3 (3)	145 (36)
ART – known donor or own gametes	51 (10)	2 (2)	48 (12)
Surrogacy – own gametes	44 (9)	42 (46)	1 (<1)
Surrogacy – unknown donor	23 (5)	23 (25)	0 (0)
Surrogacy – known donor	10 (2)	10 (11)	0 (0)

a.Includes data from 5 children with other gendered parents and 4 children where parent gender was not identified.

b.Multiple responses possible.

c.Assisted Reproductive Technology.

### Child health and wellbeing

Common childhood conditions were recorded, as well as breastfeeding data and current immunisation status. Child health was measured using three scales. The Child Health Questionnaire (CHQ), for children aged 5-17 years, and the complementary Infant Toddler Quality of Life survey (ITQOL), for children aged 0-4 years, were used to measure multidimensional aspects of functioning and health-related quality of life [43,44]. These instruments produce scores from 0-100 for child health across a number of scales, with higher scores representing better health and/or wellbeing.

The Strengths and Difficulties Questionnaire (SDQ) is a brief behavioural screening questionnaire with five scales for children aged 3-17 years [45]. Individual scale scores range from 0-10, with a total difficulties score ranging from 0-40 (excluding the prosocial scale). A lower score indicates better social and emotional wellbeing, with the exception of the prosocial scale where a higher score indicates better social and emotional wellbeing.

### Perceived stigma

Measures of perceived stigma were based on the stigmatisation scale for lesbian-parent families developed by Bos et al, the Bos Stigmatisation Scale (BSS) [18]. This was adapted to represent all same-sex attracted parents. Parents were asked to indicate how often in the past year their family had experienced stigma related to the their same-sex attraction (eg have people gossiped about you and your family, have people excluded you and your family?). Each of the seven items is scored from 1 (never) to 3 (regularly) with the final score being the mean of all items. A higher score represents more frequent experiences of perceived stigma. Internal consistency of the adapted scale was good and compared favourably to the internal consistency in its original setting (Cronbach’s α = 0.76 vs Cronbach’s α = 0.72) [18].

### Data management and analysis

Online surveys were automatically recorded into a database during survey completion and then exported into Microsoft Excel for Mac, version 14.0.2. Paper surveys were double entered into the spreadsheet for cleaning and scoring. Initially, descriptive statistics were used to describe health and wellbeing. For the CHQ and SDQ complete normative datasets were available for comparison from the Health of Young Victorians Survey (HOYVS) and the Victorian Child Health and Wellbeing survey (VCHWS) respectively [39,40].

### The health of young victorians survey (HOYVS)

The HOYVS was a school-based epidemiological study of the health and wellbeing of children aged 5-18 years conducted to provide Australian normative data for the CHQ and establish its reliability and validity in the Australian context [39]. A two stage stratified design selected 24 primary and 24 secondary schools across Victoria, Australia, within each educational sector followed by the random sampling of an entire class at each year level in each school. Parents completed a paper version of the Authorised Australian Adaptation of the CHQ between July and November 1997 for a total of 5414 children (response 72%).

### The victorian child health and wellbeing survey (VCHWS)

The VCHWS collected data on 5025 randomly selected Victorian children aged under 13 years by parent interview between February and May 2009 (response 75%) [40]. Participants were recruited using random digit dialing and were stratified by geographical distribution. Data were collected via a computerised assisted telephone interview with only one child per household included in the survey. The Strengths and Difficulties Questionnaire formed one component of the survey.

Each of the scale scores for child health from the CHQ and SDQ were used as dependent variables in mixed effects linear regression models to compare the independent variable ‘sample’ (ACHESS data or HOYVS/VCHWS normal population datasets), adjusting for socio-demographic characteristics as fixed effects predictors, while family clustering was included as a random effects predictor (see Table 2). The categorical binary variable ‘sample’ was included in the model with the ACHESS data allocated as 1 and either the HOYVS or VCHWS data allocated as 2 for each of the CHQ and SDQ. The output from the model, β, represents the difference between the ACHESS scale scores and either the HOYVS or VCHWS scale scores.

**Table 2:** Summary of scale score comparisons between the ACHESS and population data from the HOYVS (CHQ) and VCHWS (SDQ) using mixed effects multiple linear regression models

**Scale**		**β (95% CI)**	**P value**
CHQ^**a**^	Physical functioning	0.78 (-1.79, 3.35)	.55
	Role-emotional/behavioral	-1.22 (-4.02, 1.57)	.39
	Role-physical	-0.89 (-3.60, 1.81)	.52
	Bodily pain	-1.70 (-4.60, 1.20)	.25
	General behavior	2.93 (0.35, 5.52)	.03
	Mental health	-0.85 (-3.05, 1.35)	.45
	Self esteem	1.13 (-1.56, 3.82)	.41
	General health	5.60 (2.69, 8.52)	<.001
	Parental impact-emotional	0.16 (-2.92, 3.24)	.92
	Parental impact-time	-1.45 (-3.96, 1.05)	.23
	Family activities	0.38 (-3.22, 2.46)	.79
	Family cohesion	6.01 (2.84, 9.17)	<.001
SDQ^**b**^	Emotional symptoms	-0.02 (-0.23, 0.28)	.91
	Conduct problems	-0.05 (-0.29, 0.18,	.65
	Hyperactivity/inattention	-0.10 (-0.49, 0.29)	.61
	Peer problems	-0.01 (-0.27, 0.25)	.93
	Prosocial	-0.03 (-0.30, 0.24)	.83
	Total difficulties	-0.20 (-1.05, 0.65)	.65

^a^Children aged 5-18 years; adjusted for fixed effects predictors of child’s gender, child’s age, biological child, parent’s age, parent’s gender, parent’s country of birth, in a relationship, parent’s education, and parent’s employment status, and the random effects predictor of family.

^b^Children aged 4-12 years; adjusted for fixed effects predictors of child’s gender, child’s age, biological child, parent’s age, in a relationship, parent’s education, and household income, and the random effects predictor of family.

To determine whether there was an association between stigma and child health each of the scale scores from the CHQ, SDQ and ITQOL were used as dependent variables in mixed effects linear regression models with the BSS scale score as an independent continuous variable, again adjusting for socio-demographic characteristics as fixed effects predictors, with family clustering as a random effects predictor (see Table 3). The output from the model, β, represents the change in each of the scale scores for every one point increase on the BSS scale.

**Table 3:** **Summary of the relationship between BSS score and child health scale scores from the ACHESS using mixed effects multiple linear regression models**^**a**^

**Scale**	**β (95% CI)**	**P value**
*ITQOL (0-4 years)*		
Physical activity	-3.03 (-5.86, -0.21)	.04
Growth and development	-0.08 (-3.24, 3.08)	.96
Bodily pain	-7.32 (-15.07, 0.42)	.06
Temperament and mood	-2.21 (-7.40, 2.98)	.41
General behavior	-8.33 (-17.85, 1.19)	.09
Global behavior	-8.00 (-17.45, 1.43)	.10
Behavior	-5.07 (-10.72, 0.58)	.08
Combined behavior	-6.35 (-12.75, 0.06)	.05
General health	-4.76 (-11.38, 1.87)	.16
Parental impact-emotional	-3.38 (-8.46, 1.71)	.19
Parental impact-time	-1.25 (-6.32, 3.81)	.63
Family cohesion	-4.87 (-12.27, 2.52)	.20
*CHQ (5-17 years)*		
Physical functioning	-5.57 (-14.07, 2.93)	.20
Role-emotional/behavioral	-7.15 (-15.56, 1.262)	.10
Role-physical	-1.34 (-8.90, 6.21)	.73
Bodily pain	-2.60 (-12.69, 7.50)	.61
General behavior	-4.87 (-14.16, 4.42)	.30
Mental health	-10.45 (-18.48, -2.42)	.01
Self esteem	-4.04 (-15.93, 7.85)	.51
General health	2.54 (-7.72, 12.80)	.63
Parental impact-emotional	-4.50 (-13.88, 4.87)	.35
Parental impact-time	-0.20 (-9.55, 9.15)	.97
Family activities	-7.98 (-18.06, 2.10)	.12
Family cohesion	-9.82 (-17.86, -1.78)	.02
*SDQ (3-17 years)*		
Emotional symptoms	0.94 (0.08, 1.81)	.03
Conduct problems	0.39 (-0.41, 1.19)	.34
Hyperactivity-inattention	0.69 (-0.46, 1.83)	.24
Peer problems	-0.20 (-1.02, 0.62)	.63
Prosocial behavior	0.76 (-0.19, 1.72)	.12
Total difficulties	1.84 (-0.74, 4.42)	.16

^a^Data adjusted for fixed effects predictors of: parent in a relationship, parent gender, biological child, family formation (heterosexual sex, home insemination, ART, surrogacy, unknown donor), relationship at conception, parent education, household income, parent age, and region, and the random effects predictor of family.

Where appropriate, socio-demographic characteristics were dichotomised. Missing values were omitted and a significance level of 2-sided P < .05 was used. Model assumptions of normality and equality of variance were supported by appropriate residual plots. All statistical analyses were performed using STATA version 12.0.

### Ethics

All procedures were approved by The University of Melbourne Health Sciences Human Ethics Subcommittee, ethics ID number 1136875.1. All participants gave informed consent before taking part.

## Results

Three hundred and ninety eligible parents made contact with the research team and 315 completed the survey (81%), only two of which used a paper survey. These parents provided data on 500 children.

### Family structure and socioeconomic context

The socio-demographic characteristics and family structures are summarised in Table 1. Ninety-one children (18%) had a male index parent, 400 (80%) had a female index parent and 5 (1%) had an other-gendered index parent. The majority of children were living in an inner metropolitan area (261, 52%). Most children were born in Australia (427, 85%), followed by India (36, 7%) and the USA (21, 4%). Fifty-six children (11%) spoke a language other than English at home. Overall, parents had completed high levels of education with almost three quarters of children having parents who had completed a tertiary education (365, 73%). Data from the Longitudinal Study of Australian Children suggests that 28.5% of mothers with 4-8 year old children have a tertiary education [46]. Seventy-two children (79%) with a male index parent lived in households with a high combined income (over AUD $100,000), with around half of children with a female index parent living in such households (220, 55%). Median household income in Australia in 2011 was $64,168 [47].

Most children were the biological child of the index parent or of the index parent’s partner (408, 82%), with few children being fostered or adopted (15, 3%). More than two thirds of the children were born in the context of the current same-sex relationship (347, 69%), although a notable number of children were born in the context of a previous heterosexual relationship or when the parent was single (69, 14% and 33, 7% respectively). Four hundred and sixty-four (93%) children had parents who were currently in a relationship.

A comparison of key characteristics from the ACHESS with characteristics from the HOYVS and VCHWS normative datasets can be seen in Table 4. The most striking difference is in parent education level where a much higher proportion of parents from the ACHESS have a tertiary education. Both the HOYVS and VCHWS data were collected from children in Victoria. Although the ACHESS sample provides information from children across Australia, almost half (48%) live in Victoria.

**Table 4:** Comparison of key sociodemographic characteristics for the ACHESS and CHQ/SDQ normative datasets

	**CHQ (n)**		**SDQ (n)**	
	**ACHESS (219)**	**HOYVS (5355)**	**ACHESS (213)**	**SDQ (3404)**
Mean child age, years (SD)	9.41 (3.75)	11.08 (3.53)	6.88 (2.43)	8.15 (2.64)
Boys, n (%)	126 (58.06)	2727 (50.37)	114 (54.03)	1795 (52.73)
Mean parent age, years (SD)	42.43 (6.54)	39.82 (5.96)	41.07 (5.92)	40.13 (6.38)
Parents with tertiary education, n (%)	149 (68.04)	1230 (22.97)	147 (69.34)	1,195 (35.18)
Household income^1^ > $100,000, n (%)	N/A	N/A	170 (79.81)	1911 (61.57)
Single parent family, n (%)	18 (8.18)	709 (13.16)	11 (5.16)	585 (17.19)

^1^Household income not collected in the HOYVS.

### Child health and wellbeing

Asthma was the most commonly reported medical condition for all children (63, 13%). For children aged 0-14 years old the prevalence was slightly lower at 11.6%. This compares with 10% of children in this age group across Australia (2007-2008) [48]. The prevalence of medical conditions for children in the ACHESS is summarised in Table 5.

**Table 5:** Prevalence of common childhood medical conditions in the ACHESS sample

**Condition**	**Number**	**Prevalence**
Asthma	63	12.7
Dental	34	6.9
Anxiety	30	6.1
Allergies	29	5.9
Attention	28	5.7
Behavior	26	5.3
Vision	20	4.1
Learning	18	3.6
Sleep	16	3.2
Speech	15	3.1
Development	11	2.2
Orthopedic/joint	9	1.8
Depression	8	1.6
Chronic respiratory	6	1.2
Hearing	5	1.0
Diabetes	0	0
Epilepsy	0	0

The proportion of children from the ACHESS who were fully immunised at ages 1, 2 and 5 remained constant at 93%. This compared with 92%, 93% and 89% respectively for all children in Australia (2011) [48]. Eighty percent of children under the age of five years from the ACHESS were breastfed at some point. For children with a female index parent this figure was 96% and for children with a male index parent it was 22%. This compares to 90% of all Australian children who were initiated with breastfeeding (2010) [48]. The proportion of children under five years from the ACHESS who were still breastfeeding at four months was 33% (5% for children with a male index parent and 40% for children with a female index parent). This compares to 39% of children in the general population who were exclusively breastfeeding at 4 months [48].

The overall child health and wellbeing scores from the ITQOL, CHQ and SDQ for boys and girls from the ACHESS are presented in Table 6. After adjusting for socio-demographic characteristics there were no differences in SDQ scale scores for children aged 4-12 years when compared to population data (Table 2).

**Table 6:** Summary of the ACHESS scale scores (CHQ, ITQOL and SDQ) for boys and girls

**Scale**	**Mean scale score (SD)**	
	**Boys**	**Girls**
*ITQOL (0-4 years)*	*N = 112-138*	*N = 104-138*
Physical activity	98.26 (7.06)	97.90 (5.60)
Growth and development	96.99 (7.95)	98.22 (5.53)
Bodily pain	77.63 (17.05)	79.62 (16.06)
Temperament and mood	80.83 (12.05)	83.06 (10.37)
General behavior	77.08 (18.06)	74.98 (18.39)
Global behavior	86.74 (18.39)	85.82 (18.24)
Behavior	80.45 (11.13)	82.19 (9.67)
Combined behavior	79.81 (12.72)	79.93 (11.55)
General health	82.56 (15.11)	85.01 (12.61)
Parental impact-emotional	88.74 (10.06)	90.56 (10.60)
Parental impact-time	92.85 (12.15)	94.38 (9.98)
Family cohesion	87.68 (17.22)	86.05 (17.62)
*CHQ (5-17 years)*	*N = 125-126*	*N = 91-92*
Physical functioning	96.34 (13.43)	94.26 (16.51)
Role-emotional/behavioral	91.27 (17.98)	96.01 (11.94)
Role-physical	95.24 (14.43)	95.65 (14.17)
Bodily pain	84.32 (17.34)	79.35 (18.62)
General behavior	71.61 (17.79)	78.08 (15.11)
Mental health	82.08 (15.95)	82.43 (14.00)
Self esteem	80.09 (23.76)	86.68 (17.52)
General health	83.36 (18.26)	85.82 (16.72)
Parental impact-emotional	80.20 (17.97)	82.47 (15.73)
Parental impact-time	90.00 (15.55)	90.22 (15.26)
Family activities	83.90 (20.44)	87.50 (14.23)
Family cohesion	81.56 (19.39)	85.27 (14.85)
*SDQ (3-17 years)*	*N = 161*	*N = 138*
Emotional symptoms	1.66 (2.00)	1.56 (1.77)
Conduct problems	1.60 (1.64)	1.26 (1.52)
Hyperactivity-inattention	3.38 (2.51)	2.57 (2.30)
Peer problems	1.61 (1.89)	1.20 (1.38)
Prosocial behavior	7.81 (2.07)	8.27 (1.86)
Total difficulties	8.25 (5.98)	6.59 (4.72)

On the CHQ, after adjusting for socio-demographic characteristics, the overall mean score for general behaviour, general health and family cohesion was 3%, 6% and 6% higher respectively for children from the ACHESS compared to population data (β = 2.93, 95% CI = 0.35 to 5.52, P = .03; β = 5.60, 95% CI = 2.69 to 8.52, P = <.001; and β = 6.01, 95% CI = 2.84 to 9.17, P = <.001 respectively). There were no significant differences identified for other CHQ scales (Table 2).

### Perceived stigma

For two thirds of children (333, 67%) parents reported perceived stigma on at least one item of the BSS. The mean score on the BSS for all children from the ACHESS was 1.25 (SD 0.28). Perceived stigma was associated with a worse score on the physical activity scale of the ITQOL (P = .04), the mental health and family cohesion scales on the CHQ (P = .01 and P = .02), and the emotional symptoms scale on the SDQ (P = .03). The combined behavior scale on the ITQOL also approached significance (P = .052) (Table 3).

## Discussion

This is the first study of child health in same-sex parented families in Australia and the largest study of its kind internationally, to date. As such it can be used to understand a broad range of families where at least one parent is same-sex attracted. The findings suggest that there is no evidence to support a difference in parent-reported child health for most measures in these families when compared to children from population samples, which was also found with the previous smaller studies and those of lesbian families [4,18,49]. The ACHESS makes a significant contribution to the literature as it succeeded in representing children being raised by same-sex attracted parents from a broader range of family contexts than studies previously. The recruitment of 91 children with male same-sex attracted parents allows for the first time a sample size large enough to enable analysis of child health and wellbeing that includes children growing up with at least one gay male parent. Eleven per cent of children in same-sex couple households had male parents when the Australian census measured in 2011 [1], and as this number grows it is necessary to better understand child health and wellbeing in this context.

Socio-demographically, the parent sample has a high level of education and income, relative to population median income [47], and normative samples. While there is evidence to suggest that maternal education in particular is related to improved child health [50] it is not clear how this translates to same-sex families where the relationship between gender roles and parenting is less clear [30]. This difference in education and income must be considered however when viewing these results, even having adjusted for disparities in statistical analyses. Higher relative income in same-sex families is not surprising however, given that there is often a need to engage in costly and complex medical procedures in order to create a family where the parents are same-sex attracted. Children with male index parents are more commonly born through surrogacy arrangements. However, with commercial surrogacy illegal throughout Australia, and altruistic surrogacy poorly established, these arrangements often take place overseas, and thus parents with lower incomes may be less likely to avail this method. This situation also explains the number of children with a male index parent who were born in the US and India, two of the more commonly accessed territories for this process. Further, despite the fact that Australia is yet to have legislated to ensure marriage equality it appears that family transitions in our sample of same-sex parent families are similar to the general population. For our sample over two thirds of children were born in the context of their parents’ current relationship compared with 65-81% of children, depending on the age of the youngest child, for all families in Australia (2006-7) [51].

Key child health promotion and illness prevention strategies appear to resonate well with our sample of same-sex parent families as seen by the rates of immunisation and breastfeeding. Despite the lack of an easy supply, a number of our male parents strove to ensure that their children receive some breast milk in early life, with a significant proportion of these children born via surrogacy (64%). This is usually achieved via surrogate milk donation and is an indication that same-sex male parents make efforts to support this health strategy for their children.

In comparing CHQ and SDQ scale scores from the ACHESS to population normative data it is possible to understand the multidimensional aspects of child health and wellbeing in a broader context. The three areas where statistically significant differences were seen are general behavior, general health and family cohesion. Population and clinical studies have demonstrated that there are socially and clinically meaningful differences of 5 points on the scales within the CHQ. The small difference seen between the ACHESS sample and the general population on the general behaviour domain was not observed with the behavioural components of the SDQ, which may be related to the origin and development of the two measures, where the CHQ is more related to functioning and the SDQ used as a screening tool. This is an important area for further exploration. The general health scale is a broad concept measured by a single item within the CHQ, however the size of difference is notable. Qualitative interviews accompanying this study might raise themes and issues to enable greater understanding of what could be influencing this finding, possibly related to improved communication via family cohesion. The results for family cohesion are significant. Previous research has suggested that same-sex attracted parents are much more likely to share household duties equally when compared to their heterosexual counterparts, and they make decisions about work/family balance based more on circumstance than preconceived gender-based ideals [30]. Individual suitability rather than societal convention is more likely therefore to inform parenting roles. This has the potential to engender greater family harmony in the long-term.

Whilst children with same-sex attracted parents from our sample demonstrate comparable health to other children across the population, it is clear that they, and their families, are experiencing stigma. Previous work has suggested that stigma and homophobia are related to problem behavior and conduct problems in children with same-sex attracted parents [17,33,18]. Our findings support and strengthen the idea that stigma related to parental sexual orientation is associated with a negative impact on child mental and emotional wellbeing. Other family contexts have seen similar associations between stigma and child mental health including race-based stigma in Aboriginal and Torres Strait Islander families, and social stigma in single-parent families [52,53]. Stigma can be experienced in numerous social contexts, educational settings and healthcare environments. Lesbian parents in Australia have previously described the barriers they perceive when accessing healthcare services and often times have to choose a disclosure strategy to adopt when dealing with practitioners [25]. Instead of feeling accepted when seeking healthcare for their children this perceived stigma can lead to a sense of vulnerability where in fact healthcare services should “be a safe place for lesbians [and gay men] to authentically talk about their relationships with lovers, friends and family [54]”. If the negative impact of perceived stigma on child mental and emotional wellbeing is compounded in a healthcare environment, where parents do not feel free to discuss their family in its entirety, then the health ramifications for children will only be amplified.

### Limitations

Whilst the ACHESS is the largest study of its kind to date, the use of a convenience sample to access the highest number of participants needed to be worked through carefully as there are no current options to access data through regular population surveys or administrative datasets. Every effort was made to recruit a representative sample [35], and from the limited data available about same-sex parent families it appears that the ACHESS sample does reflect the general context of these families in contemporary Australia [37]. The self-selection of our convenience sample has the potential to introduce bias that could distort results. It is clear that the families from the ACHESS are earning more and are better educated than the general population. This has in part been allowed for in the statistical analysis by incorporating numerous control variables that are recognised to have an impact on child health outcomes but the results should be read with these differences in mind. If systematic bias was at play however, it would be anticipated that all outcome variables would demonstrate higher scores across the sample. As it is, only three key variables on the CHQ demonstrated significant difference, and none of the scale scores from the SDQ. Whether there are real differences between the ACHESS sample and the normative population or not, it is clear that there are aspects at play in our sample of same-sex families that allow improved outcomes in general behavior, general health, and in particular family cohesion.

Parent-report of child health also has its limitations. While parents are not able to fully understand the lived experience of their children’s health in all aspects of life it is possible to draw inferences from the data that they represent. In particular comparisons with population normative data are valid given that parent-report was used in both contexts. There is no evidence to suggest that any group of parents would systematically respond in a particular way on any given scale, however this cannot be discounted entirely. Future research will report on child-reported measures of health, as well as a contextual analysis of qualitative data drawn from family interviews, in order to draw out any bias that parental reporting might have.

## Conclusions

This study demonstrates that children with same-sex attracted parents in Australia are being raised in a diverse range of family types. These children are faring well on most measures of child health and wellbeing, and demonstrate higher levels of family cohesion than population samples. Perceived stigma is experienced by children with same-sex attracted parents, which is an issue that requires attention in all settings. In particular this is of importance to healthcare services, where parents seek assistance for their own physical and mental health, as well as that of their children. As perceived stigma already has a negative impact on mental and emotional wellbeing, negative experiences in healthcare settings will serve to exacerbate any problems that these children, and their families, might face. Future work should further explore the ways in which stigma affects the mental health of children with same-sex attracted parents and in particular ways in which these children can be protected from experiences of discrimination. Same-sex families are becoming increasingly visible globally. These results from Australia provide important epidemiological data across health, wellbeing, morbidity and perceived stigma of children with same-sex attracted parents, and an essential contemporary contribution to the current policy-research interface.

## Abbreviations

ACHESS: The Australian Study of Child Health in Same-Sex Families; CHQ: Child Health Questionnaire; ITQOL: Infant Toddler Quality of Life Survey; SDQ: Strengths and Difficulties Questionnaire; BSS: Bos Stigmatization Scale; HOYVS: Health of Young Victorians Survey; VCHWS: Victorian Child Health and Wellbeing Survey.

## Competing interests

The authors declare that they have no competing interests

## Authors’ contributions

SRC initiated the project, designed the survey instrument, implemented the survey, monitored data collection for the survey, developed the statistical analysis plan, cleaned and analysed the data, and drafted and revised the paper. He is guarantor. EW designed the survey instrument, monitored data collection for the survey, revised the paper, and supervised SRC. RM designed the survey instrument, monitored data collection for the survey, revised the paper, and supervised SRC. JP designed the survey instrument, and revised the paper. ED designed the survey, analysed the data, and revised the paper. All authors, external and internal, had full access to all the data (including statistical reports and tables) in the study and take responsibility for the integrity of the data and the accuracy of the data analysis. All authors read and approved the final manuscript.

## Pre-publication history

The pre-publication history for this paper can be accessed here:

http://www.biomedcentral.com/1471-2458/14/635/prepub
